# Effect of different filling tendencies on the spatial quantum Zeno effect

**DOI:** 10.1038/s41598-018-27605-9

**Published:** 2018-07-06

**Authors:** Xin Zhang, Chang Xu, Zhongzhou Ren, Jie Peng

**Affiliations:** 1 Department of Physics, Nanjing University, Nanjing, 210008 China; 2 School of Physics Science and Engineering, Tongji University, Shanghai, 200092 China; 3 Laboratory for Quantum Engineering and Micro-Nano Energy Technology and School of Physics and Optoelectronics, Xiangtan University, Hunan, 411105 P.R. China

## Abstract

The quantum Zeno effect is deeply related to the quantum measurement process and thus studies of it may help shed light on the hitherto mysterious measurement process in quantum mechanics. Recently, the spatial quantum Zeno effect is observed in a Bose-Einstein condensate depleted by an electron beam. We theoretically investigate how different intrinsic tendencies of filling affect the quantum Zeno effect in this system by changing the impinging point of the electron beam along the inhomogeneous condensate. Surprisingly, we find no visible effect on the critical dissipation intensity at which the quantum Zeno effect appear. Our finding shows the recent capability of combining the Bose-Einstein condensate with an electron beam offers a great opportunity for studying the spatial quantum Zeno effect, and more generally the dynamics of a quantum many-body system out of equilibrium.

## Introduction

Due to the peculiar laws of quantum mechanics, frequent enough observation can halt the evolution of a system. This is known as the quantum Zeno effect^1^. It is deeply connected to the measurement process in quantum mechanics^2^. Studies of the quantum Zeno effect may thus help shed light on the fascinating and hitherto mysterious nature of the quantum measurement process^3,4^. The quantum Zeno effect has been realized experimentally in various different physical systems^5–18^. Interestingly however, most of these experiments are performed in quantum mechanical systems involving several discrete energy levels, while the quantum Zeno effect of the continuous spatial distribution of a quantum mechanical system has been much less explored. Recently, such spatial quantum Zeno effect has been observed in a Bose-Einstein condensate^16^, where a stronger electron beam depleting the condensate counterintuitively led to less depletion.

From another angle of view, previous theoretical studies^19–24^ of the spatial quantum Zeno effect have been mainly concerned with single-particle motion, while how such effect occurs in a many-body system is less studied. The above system using a Bose-Einstein condensate offers a great opportunity to study such many-body spatial quantum Zeno effect.

Concerning this specific system, previous works^16,25,26^ have predicted the existence of the quantum Zeno effect, and have studied how the characteristics of the time evolution depends on the relation between the width of the depletion region and the speed of sound in the condensate^25^, how the magnitude of the Zeno suppression depends on the width of the depletion and the strength of interatomic interaction^16^, how the existence of the quantum Zeno effect depends on how fast the depletion intensity decays in its wings, as well as the existence of different steady-flow patterns under depletion^26^.

In the present work, we study using a time-dependent approach how the spatial quantum Zeno effect in this system depends on the intrinsic tendency of the system to fill in the depletion region.

Intuitively, if the electron beam impinges at places where the trapping potential is higher and the density is lower, for example at the wings of a harmonic trap rather than at the center, the intrinsic tendency of the system to fill in these places will be lower. From another point of view, the kinetic energies of the atoms near such impinging position would be lower. An interesting question can be raised about how this affects the quantum Zeno effect. One might naturally expect when the dissipation happens at a point with lower tendency of filling, the spatial quantum Zeno effect should be more easily observed, i.e., appear at a lower dissipation strength. Surprisingly, in our investigations except for an overall scaling factor, different impinging points show no visible effect on the spatial quantum Zeno effect, and in particular show no visible effect on the critical dissipation strength at which the quantum Zeno effect starts to appear.

## Results

### Physical system

**Figure 1:**
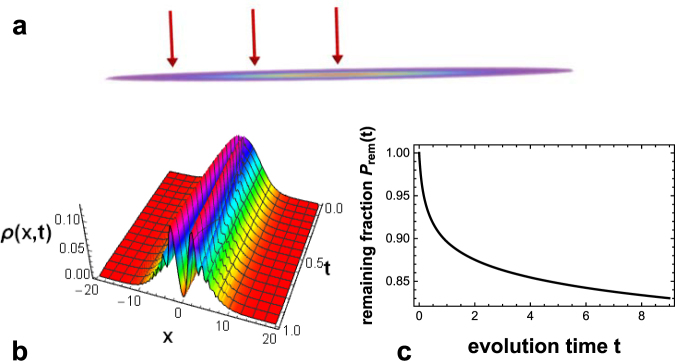
Physical system and the physical processes. (**a**) The physical system. A one-dimensional Bose-Einstein condensate is trapped in a harmonic trap. An electron beam (red arrows) knocking atoms out of the condensate can impinge at different chosen positions of the inhomogeneous condensate. (**b**) The time evolution of the density distribution *ρ*(*x*, *t*) of the condensate. The electron beam burns a hole at its impinging point *x*_*d*_ = 0 and depletes the condensate. The time evolution is calculated using equation (1) and *ρ*(*x*, *t*) = |*ψ*(*x*, *t*)|^2^. (**c**) The remaining fraction ${{\rm{P}}}_{{\rm{r}}{\rm{e}}{\rm{m}}}(t)\equiv {\int }_{-\infty }^{\infty }\,\rho (x,t)dx$ of the condensate is a decreasing function of time. In (**b** and **c**) *w* = 0.1, *γ* = 23.4. For the precise definitions of the symbols, please see Methods.

A schematic depiction of the physical setup is shown in Fig. 1a. It constitutes of a one-dimensional Bose-Einstein condensate in a harmonic trap under the action of an impinging electron beam knocking atoms out of the condensate. The impinging point of the electron beam can be changed along the one-dimensional condensate. The details of the theoretical descriptions are given in Methods.

### The spatial quantum Zeno effect

An exemplary time-evolution of the density distribution of the condensate is shown in Fig. 1b. Initially, the condensate is in the ground state. The electron beam acts as a sink for atoms and burns a hole at the impinging position. Due to the depletion from the electron beam, the remaining fraction of the condensate is a decreasing function of time, as shown in Fig. 1c.

**Figure 2:**
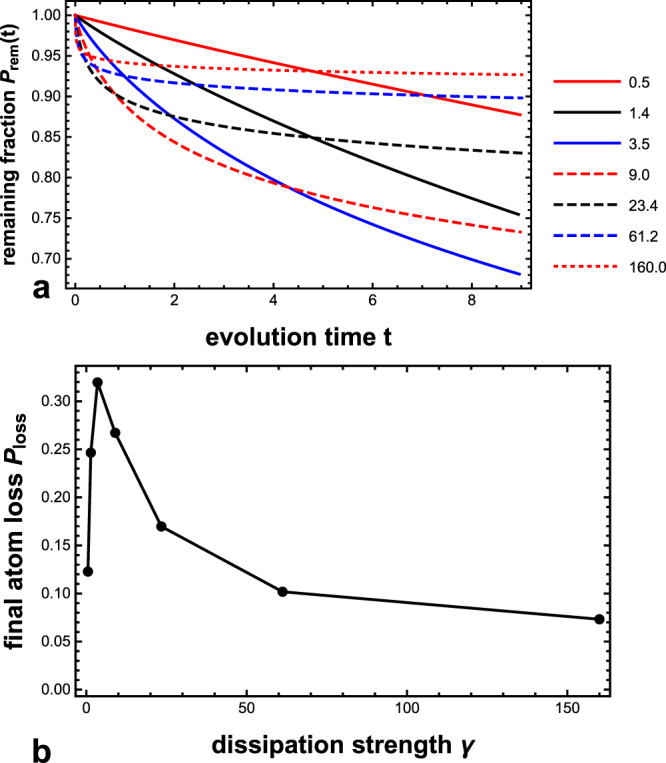
The spatial quantum Zeno effect. (**a**) The remaining fraction of the condensate *P*_rem_(*t*) as a function of time, for different intensities of the electron beam. The spatial quantum Zeno effect occurs: starting from the intensity parameter *γ* = 9.0, as the dissipation further increases, the final remaining fraction increases rather than decreases. (**b**) The total atom loss at the end of evolution *P*_loss_ as a function of the dissipation-intensity parameter *γ*. The quantum Zeno effect is manifested as the decreasing part of the curve. *w* = 0.1, *x*_*d*_ = 0. For the precise definitions of the symbols, please see Methods.

The spatial quantum Zeno effect can be observed in this system^16,25,26^. For example (Fig. 2a), for the width parameter *w* = 0.1 (cf. equation (3)), as the intensity of the electron beam becomes larger, the total loss initially rises, but then decreases as the dissipation increases further. This is the quantum Zeno effect. This can be seen more simply (Fig. 2b) by plotting the total atom loss at the end of evolution as a function of the dissipation strength parameter, namely the intensity parameter of the electron beam *γ* (cf. equation (3)). The existence of the decreasing part of this curve is the quantum Zeno effect.

### Influence of different filling tendencies on the spatial quantum Zeno effect

**Figure 3:**
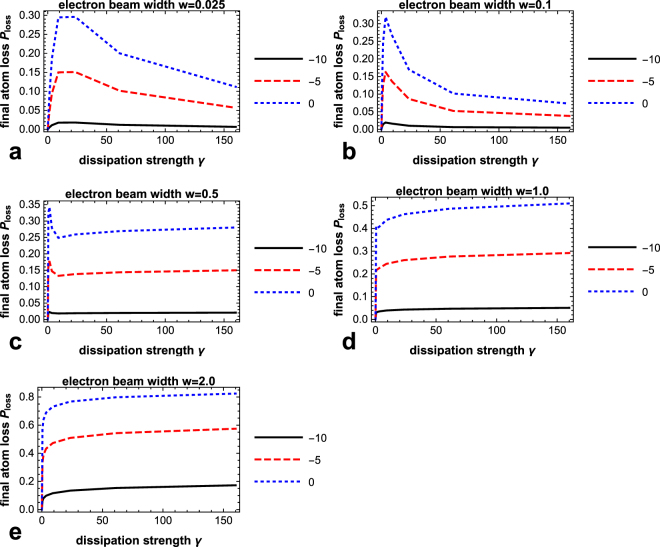
Influence of different tendencies of filling on the spatial quantum Zeno effect. (**a**) Final atom loss *P*_loss_ as a function of the dissipation-strength parameter *γ*, for three different impinging points of the electron beam: at the center of the condensate *x*_*d*_ = 0 (dotted), at the wing *x*_*d*_ = −5 (dashed), and further out in the wing *x*_*d*_ = −10 (solid). The width parameter *w* = 0.025. Different impinging points have no visible effect on the starting value of *γ* for the quantum Zeno effect, i.e. the value at which the curve starts to go downward, but only adds an overall scaling factor to the curve. (**b**–**e**) Same as (**a**) but for another four different widths *w* = 0.1, 0.5, 1.0, 2.0 of the electron beam. In line with previous studies, different beam widths affect the shapes of the curves significantly. Remarkably, for all these different beam widths, different impinging points always simply rescale the curve but has no visible effect on the critical value of the dissipation strength at which quantum Zeno effect sets in. For the precise definitions of the symbols, please see Methods.

To investigate how the spatial quantum Zeno effect will be affected by different intrinsic tendencies of filling, we run the simulation with the electron beam impinging at different positions of the inhomogeneous condensate. The results are given in Fig. 3.

In Fig. 3a, the total loss as a function of the dissipation intensity are shown for three different impinging points, represented by the black solid, red dashed and blue dotted curves respectively. Surprisingly, while changing the impinging point of the electron beam affects the absolute magnitude of the atom loss, it has no visible effect on the critical dissipation intensity at which the spatial quantum Zeno effect starts to show up. In fact, the shape of the curves for different impinging points are very similar to each other except for an overall scaling factor.

In Fig. 3b–e, the same simulations are run for another four different width parameters *w* of the electron beam. In line with previous studies, the shape of the curves are affected significantly by different width of the electron beam. For a narrow electron beam, the quantum Zeno effect is more conspicuous (Fig. 3a). For wider electron beams, the critical Zeno intensity shifts to lower values (Fig. 3b) and for even wider beams (Fig. 3d–e), the loss becomes a monotonically rising function of the dissipation strength and the quantum Zeno effect is no longer visible. Remarkably, for all the widths investigated corresponding to Fig. 3a–e, different impinging points have no visible effect on the critical Zeno intensity or the shape of the curves, but only scales the atom losses by an overall factor. The details of our calculations are given in Methods.

## Discussion

In this paper we investigate the effect of different intrinsic tendencies of filling on the spatial quantum Zeno effect by varying the impinging point of the electron beam along the inhomogeneous Bose-Einstein condensate. Surprisingly, impinging at different densities do not change the critical dissipation intensity at which the quantum Zeno effect starts to appear, but just adds an overall scaling factor to the atom loss as a function of the dissipation intensity.

Since to reach the Zeno regime is required in protocols employing the quantum Zeno effect for quantum state engineering, to reliably estimate at what dissipation strength the quantum Zeno effect sets in is very useful. So the invariance of the critical Zeno strength with respect to different filling tendencies as observed in our work may also be of practical interest. The explanation for this invariance invites further study.

We offer a probable explanation for this invariance as follows. The essence of the Zeno suppression is that measurement is repeated frequent enough that the coherent evolutions are halted. In our scenario, a higher dissipation strength corresponds to a higher repetition frequency. How high a repetition frequency is sufficient for the onset of the Zeno suppression is determined by the time scale of the coherent evolution, and consequently by its energy scale. A probable explanation of the observed invariance is thus that, in the major part of the evolution a quasi-equilibrium has been maintained and consequently the energy scales at different points of the inhomogeneous condensate are the same, as a result of which the critical dissipation strengths for the onset of Zeno suppression for these points are the same. This explanation, if confirmed, would mean that the critical Zeno strength can be used as a sensitive prob for the local energy scale, which may be used as a new tool for studying complex systems at and out of equilibrium.

In our investigation, we have used the Gross-Pitaevskii equation (cf. Methods), which is a mean-field equation. Due to its extensive verification from experiments, we believe its predictions are reliable. In future investigations, to take into account the full quantum many-body character of the problem would be a fascinating topic to pursue.

The fact that the Zeno suppression can be observed for high enough bombardment rates can be justified as follows: Apart from the dissipation, at each instant of time the evolution of the system is governed by a Hamiltonian ${H}_{{\rm{inst}}}=-\,\frac{{\hslash }^{2}}{2m}\frac{{\partial }^{2}}{\partial {x}^{2}}+g|\psi (x,t){|}^{2}+V(x)$ (cf. equation (1) and texts thereafter). This means at high dissipation strength the standard argument for the Zeno effect also applies. The continuous dissipation can be viewed effectively as a periodic process. Within each short repetition period *δt*, starting from zero probability distribution inside the dissipation region, the wave function of the condensate first evolves according to *ψ*(*t*_0_) → *ψ*(*t*_0_ + *δt*) = exp (−*iH*_inst_*δt*)*ψ*(*t*_0_). This gives a probability outside the dissipation region (~1 − *c*(*t*)*δt*^2^), that is, a change second order in time, where *c*(*t*) is a small positive number. Subsequently due to fast dissipation, any wave amplitude in the dissipation region is quickly nulled and the evolution starts afresh. For higher bombardment rates, the effective repetition time *δt* becomes smaller. After a fixed time *t* the probability for the system to remain outside the dissipation region is ~(1 − *cδt*^2^)^*t*/*δt*^, which goes to unity as *δt* becomes small. (For this estimation we have used a time-averaged *c* instead of *c*(*t*). This would not alter the conclusion.) This means the atom loss goes to zero as the bombardment rate becomes large, i.e., the Zeno suppression happens.

As can be seen from Figs 2b and 3, the atom loss initially rises as the dissipation increases. As a general trend, this is in line with expectation: a zero dissipation would lead to no atom loss, while increasing the dissipation from zero should naturally increase the atom loss. Nevertheless, there may be an interesting phenomenon hidden beneath, namely the anti-Zeno effect, i.e., accelerated decay due to frequent measurements. The quantum Zeno and anti-Zeno effect were predicted by pioneering works^27–29^ and have been experimentally confirmed in^7,8^. Also, both the quantum Zeno and anti-Zeno effect have been predicted for a Bose-Einstein condensate in a quantum many-body description^30–32^. In the experiments in^7,8^, the measurements itself do not cause dissipation, while in our scenario the dissipation *is* the measurements. Consequently the anti-Zeno effect may be inherently intertwined with dissipation. It can be that the anti-Zeno effect actually plays a major role in the initial rise of the atom loss with dissipation. It would be interesting to find out.

Our finding shows the recent capability of combining the Bose-Einstein condensate with an electron beam offers a great opportunity for studying the spatial quantum Zeno effect, and the dynamics of a quantum many-body system out of equilibrium.

## Methods

The evolution of the system is governed by the time-dependent Gross-Pitaevskii equation with a dissipation term^16,25,26^:

1 $$i\frac{\partial \psi (x,t)}{\partial t}=-\,\frac{{\hslash }^{2}}{2m}\frac{{\partial }^{2}}{\partial {x}^{2}}\psi (x,t)+g|\psi (x,t{)|}^{2}\psi (x,t)+V(x)\psi (x,t)-i{\rm{\Gamma }}(x)\psi (x,t\mathrm{).}$$

Here *ψ*(*x*, *t*) is the wave function of the Bose-Einstein condensate, *g* is the nonlinearity parameter arising from atom-atom interaction. In this work we have used *g* = 0.1. *V*(*x*) = *v*_*h*_*x*^2^ is the harmonic trapping potential. Without loss of generality, we have set *m* = *ℏ* = 1, *v*_*h*_ = 0.0005, and normalized the initial *ψ*(*x*, 0) such that

2 $${\int }_{-\infty }^{\infty }|\psi (x{\mathrm{,0)|}}^{2}=1.$$

The dissipation due to the impinging electron beam is described by

3 $${\rm{\Gamma }}(x)=\gamma {e}^{-{(\frac{x-{x}_{d}}{w})}^{2}}.$$

where *γ* characterizes the intensity of the electron beam and thus the intensity of the dissipation, *w* characterizes the width of the electron beam while *x*_*d*_ is the coordinate of the center of the beam.

The initial wave function is got by solving for the ground state of the time-independent Gross-Pitaevskii equation without dissipation:

4 $$\mu {\psi }_{0}=-\,\frac{{\hslash }^{2}}{2m}\frac{{d}^{2}}{d{x}^{2}}{\psi }_{0}+g|{\psi }_{0}{|}^{2}{\psi }_{0}+V(x){\psi }_{0},$$

where *μ* is the chemical potential.

In the numerical calculations, we first find the ground state of equation (4) using the optimal damping algorithm^33^ as follows.

Define

5 $${H}_{0}=-\,\frac{{\hslash }^{2}}{2m}\frac{{d}^{2}}{d{x}^{2}}+V(x),$$

and a Hamiltonian as a function of density

6 $$H(\rho )=-\,\frac{{\hslash }^{2}}{2m}\frac{{d}^{2}}{d{x}^{2}}+V(x)+g\rho (x\mathrm{).}$$

Set a very small positive number, like 10^−10^ as the convergence tolerance ${\epsilon }_{tol}$. Start from a random, normalized *ψ*_1_(*x*), compute *ρ*_1_(*x*) = |*ψ*_1_(*x*)|^2^, *f*_1_ = 〈*ψ*_1_|*H*_0_|*ψ*_1_〉, and *h*_1_ = 〈*ψ*_1_|*H*(*ρ*_1_)|*ψ*_1_〉.

Next perform the iterations. Before each iteration, one has the knowledge of *ρ*_1_, *f*_1_ and *h*_1_ from previous iteration or from initialization. Each iteration proceeds as follows.

First, calculate the normalized ground state of *H*(*ρ*_1_), denote it as ${\psi ^{\prime} }_{1}$ and perform the following calculations:

7 $$\begin{array}{ccc}{f{\rm{^{\prime} }}}_{1} & = & \langle {\psi {\rm{^{\prime} }}}_{1}|{H}_{0}|{\psi {\rm{^{\prime} }}}_{1}\rangle ,\\ {h{\rm{^{\prime} }}}_{1} & = & \langle {\psi {\rm{^{\prime} }}}_{1}|H({\rho }_{1})|{\psi {\rm{^{\prime} }}}_{1}\rangle ,\\ {\rho {\rm{^{\prime} }}}_{1}(x) & = & |{\psi {\rm{^{\prime} }}}_{1}(x{)|}^{2},\\ {h{\rm{^{\prime} }}{\rm{^{\prime} }}}_{1} & = & \langle {\psi {\rm{^{\prime} }}}_{1}|H({\rho {\rm{^{\prime} }}}_{1})|{\psi {\rm{^{\prime} }}}_{1}\rangle ,\\ {s}_{1} & = & {h{\rm{^{\prime} }}}_{1}-{h}_{1},\\ {c}_{1} & = & {h}_{1}+{h{\rm{^{\prime} }}{\rm{^{\prime} }}}_{1}-2{h{\rm{^{\prime} }}}_{1}+{f{\rm{^{\prime} }}}_{1}-{f}_{1},\\ {\alpha }_{1} & = & -{s}_{1}/{c}_{1}.\end{array}$$

Second, if *α*_1_ ≥ 1, set *α*_1_ = 1, otherwise leave *α*_1_ as it is. Then perform the following calculations:

8 $$\begin{array}{ccc}{E}_{{\rm{o}}{\rm{d}}{\rm{a}}} & = & \frac{1}{2}({f}_{1}+{h}_{1})+{\alpha }_{1}{s}_{1}+\frac{{\alpha }_{1}^{2}}{2}{c}_{1},\\ {\rho }_{2} & = & (1-{\alpha }_{1}){\rho }_{1}+{\alpha }_{1}{\rho {\rm{^{\prime} }}}_{1},\\ {f}_{2} & = & (1-{\alpha }_{1}){f}_{1}+{\alpha }_{1}\,{f{\rm{^{\prime} }}}_{1},\\ {h}_{2} & = & 2{E}_{{\rm{o}}{\rm{d}}{\rm{a}}}-{f}_{2}.\end{array}$$

Set the values of *ρ*_1_, *f*_1_ and *h*_1_ to *ρ*_1_ = *ρ*_2_, *f*_1_ = *f*_2_ and *h*_1_ = *h*_2_. If $|\frac{{s}_{1}}{{E}_{{\rm{oda}}}}| > {\epsilon }_{tol}$, repeat the iteration. Otherwise, stop the iterations. ${{\psi }^{\prime} }_{1}$ is the desired ground state.

Then using this as the initial condition, we time-integrate equation (1) to study the evolution. In the numerical implementation of the time-dependent evolution, since there can be a large separation of length scale between the sharp electron beam and the whole condensate, the spatial grid has to be dense while its extent has to be large. Because of this, to ensure convergence we had to use a spatial grid of ~160000 points. Also due to the large dissipation strength needed for quantum Zeno effect, the partial differential equation can be quite stiff numerically. Both of these in combination make the computation fairly time consuming.

### Data availability

The datasets generated during the present study are available from the corresponding author (C.X.) upon reasonable request.
